# Do sex, age, and comorbidities modify the association of socioeconomic status and opioid use after total hip arthroplasty?: a population-based study from the Danish Hip Arthroplasty Register

**DOI:** 10.2340/17453674.2024.40708

**Published:** 2024-05-17

**Authors:** André S KLENØ, Inger MECHLENBURG, Maaike G J GADEMAN, Henrik T SØRENSEN, Alma B PEDERSEN

**Affiliations:** 1 Department of Clinical Epidemiology, Department of Clinical Medicine, Aarhus University and Aarhus University Hospital, Aarhus, Denmark; 2 Department of Orthopaedic Surgery, Department of Clinical Medicine, Aarhus University Hospital and Aarhus University, Aarhus, Denmark; 3 Department of Orthopaedics, Department of Clinical Epidemiology, Leiden University Medical Center, the Netherlands

## Abstract

**Background and purpose:**

We aimed to examine the association between socioeconomic status (SES) markers and opioid use after primary total hip arthroplasty (THA) due to osteoarthritis, and whether sex, age, or comorbidities modify any association.

**Methods:**

Using Danish databases, we included 80,038 patients undergoing primary THA (2001–2018). We calculated prevalences and prevalence ratios (PRs with 95% confidence intervals [CIs]) of immediate post-THA opioid use (≥ 1 prescription within 1 month) and continued opioid use (≥ 1 prescription in 1–12 months) among immediate opioid users. Exposures were individual-based education, cohabitation, and wealth.

**Results:**

The prevalence of immediate opioid use was ~45% in preoperative non-users and ~60% in preoperative users (≥ 1 opioid 0–6 months before THA). Among non-users, the prevalences and PRs of continued opioid use were: 28% for low vs. 21% for high education (PR 1.28, CI 1.20–1.37), 27% for living alone vs. 23% for cohabiting (PR 1.09, CI 1.04–1.15), and 30% for low vs. 20% for high wealth (PR 1.43, CI 1.35–1.51). Among users, prevalences were 67% for low vs. 55% for high education (1.22, CI 1.17–1.27), 68% for living alone vs. 60% for cohabiting (PR 1.10, CI 1.07–1.12), and 73% for low wealth vs. 54% for high wealth (PR 1.32, CI 1.28–1.36). Based on testing for interaction, sex, age, and comorbidity did not statistically significant modify the associations. Nevertheless, associations were stronger in younger patients for all SES markers (mainly for non-users).

**Conclusion:**

Markers of low SES were associated with a higher prevalence of continued post-THA opioid use. Age modified the magnitude of the associations, but it was not statistically significant.

Opioid overprescribing after elective surgery has been observed in general [1] and after total hip arthroplasty (THA) surgery for osteoarthritis (OA) [2]. The role of socioeconomic status (SES) and its impact on opioid use after surgery have received little attention, even though social inequality is an increasingly recognized problem [3]. 2 studies have shown an approximately 3-fold risk of persistent opioid use in persons with low vs. high SES after adjustment for potential confounders [3]. However, both studies were conducted in a background population with patient characteristics that differ from those of the THA population.

Recently, we reported social inequality in analgesic use. The prevalence of overall analgesic use before and after THA was higher among those who had low income or low education or were living alone compared with patients who had a high income or high education or were cohabiting [4]. However, the study was mainly exploratory and did not include analytical analyses of the association between SES and opioid use after THA.

Based on the literature, we hypothesized that sex and age might modify the association between SES and opioid use due to reported differences in THA outcomes [5] and pain perception [6,7]. Low SES is also related to high prevalence of physical and mental comorbidities [8,9]. The interplay among SES, sex, age, comorbidity, and opioid use after primary THA has not been examined previously.

We aimed to examine the association between SES markers and opioid use after primary THA for OA and whether sex, age, or comorbidities modify any association.

## Methods

### Study design and population

We conducted a population-based cohort study in Denmark using medical and administrative databases. Denmark has a source population of ~5.8 million (2018), and all residents receive a unique civil personalized registration number (CPR number) at birth or upon immigration, enabling individual-level linkage of data across multiple databases [10]. The study cohort included all Danish patients undergoing primary THA during 2001–2018. Patients were identified from the Danish Hip Arthroplasty Register, which contains information on THA surgeries from all public orthopedic departments and private hospitals in the country. The Danish Hip Arthroplasty Register has a completeness of more than 95% and high accuracy of data registration [11,12].

### Socioeconomic status markers

We included data on education and income, which are 2 of the most commonly analyzed aspects of SES when evaluating health inequalities. Social networks and support have been recognized as important social determinants of health in elderly people, so we included cohabitation as an additional SES marker. Individual-level information on education and income was collected from the Population Education Register and the Income Statistics Register. Education level was based on the highest obtained education and categorized as low (primary education or lower secondary education), medium (vocational education and training, qualifying educational programs, upper secondary education, or short cycle tertiary education), or high (bachelor’s programs, master’s programs, and PhD programs or higher). Wealth was mapped from 2 variables based on the patient’s age. For patients aged < 65 years we used family income, whereas for patients aged ≥ 65 years we used family liquid assets. Residents in Denmark usually retire around the age of 65, and income would not be a valid measure for residents who receive a state pension, while family liquid assets do not apply to those who are in their very early working career. To account for yearly variation, both family income and family liquid assets were based on an average of the last 5 years leading up to the year of THA. Family income and family liquid assets were divided into 3 equally large groups and categorized as low, medium, or high income and liquid assets. Wealth was defined as low, medium, or high based on the combination of the respective groups of income and liquid assets. Information on cohabitation up to THA was obtained from the Civil Registration System [10] and defined as living alone or cohabiting (married or living with a partner).

### Opioids

We used the Danish National Prescription Registry to obtain information on opioid use based on Anatomical Classification System (ATC) codes and dispensation dates [13] (Table 1, see Appendix). The registry contains information on all prescriptions redeemed by Danish residents in community pharmacies since 1995 (excluding hospital dispensations).

**Table 1:** ATC codes for included medications

Name	ATC code
Opioids	
Morphine	N02AA01
Hydromorphone	N02AA03
Nicomorphine	N02AA04
Oxycodone	N02AA05
Oxycodone + naloxone	N02AA55
Pethidine	N02AB02
Fentanyl	N02AB03
Buprenorphine	N02AE01
Ketobemidone & antispasmodics	N02AG02
Codeine & paracetamol	N02AJ06
Tramadol	N02AX02
Tapentadol	N02AX06
Methadone	N07BC02
Codeine	R05DA04
Psychiatric medication	
Any psychiatric medication	N05, N06
NSAIDs	
Any NSAIDs	M01A

Patients were considered as preoperative non-users if they had not redeemed any opioid prescriptions in the 0–6 months before their THA. We considered 6 months to be an appropriate interval because it was highly unlikely that discontinued opioid treatment initiated > 6 months before THA would be related to any current OA-related pain. If patients redeemed at least 1 prescription for opioids during the same time interval, they were considered preoperative users.

### Potential effect measure modifiers

Information on sex and age at the time of THA was available from the Civil Registration System. We collected information on comorbidities and scored them using the Charlson Comorbidity Index (CCI). Furthermore, we identified preoperative psychiatric medication and nonsteroidal anti-inflammatory drugs (NSAIDs) to cover additional aspects of comorbidities. We used the Danish National Patient Registry to obtain a 10-year history of hospitalizations and inpatient and outpatient clinic visits prior to THA [14]. This registry contains data on all hospital admissions since 1977 and all outpatient clinic and emergency visits since 1995, coded using the International Classification of Diseases (Eighth Revision until the end of 1993 and Tenth Revision thereafter) adapted for administrative purposes. We computed a CCI score for each patient based on 19 disease categories. We defined 3 levels of CCI scores: low (0), medium (1–2), and high (≥ 3). Psychiatric hospital contacts are not included in the CCI, and we obtained data on psychiatric medication and NSAID use 6 months before THA from the Danish National Prescription Registry [13] to roughly define (i) psychiatric disorders such as mild depression or anxiety and (ii) less severe musculoskeletal disorders (including less severe OA) treated by general practitioners only. Patients were classified as preoperative non-users of psychiatric medication and NSAIDs if they had not redeemed at least 1 of the respective prescriptions during 0–6 months before THA; if they had done so in that period, they were classified as users of these medications. All ATC codes are presented in Table 1 (see Appendix).

### Statistics

Patient characteristics by SES markers were described for our study cohort. We computed the prevalence of patients who used opioids for time intervals of 0–1, 1–3, 3–6, 6–9, and 9–12 months after THA. Patients were considered opioid users in each of the intervals if they redeemed at least 1 prescription for opioids.

For immediate opioid use (within 1 month after THA) and continued opioid use (1–12 months after THA), we calculated prevalences, prevalence differences, and prevalence ratios (PRs). Both analyses were based on redeeming at least 1 opioid prescription and while immediate opioid use was based on all patients who underwent THA, continued opioid use was based on the subpopulation of immediate opioid users. Crude prevalence differences were calculated by subtracting the prevalence of high SES markers from the prevalence of low SES markers (for example, subtracting the prevalence of opioid use in patients who were cohabiting from the prevalence in patients living alone). Using binomial regression, we further calculated prevalence differences adjusted for sex and age. PRs were calculated using log-binomial regression and presented as both crude estimates and adjusted for sex and age. All analyses on immediate and continued opioid use were presented for education (with high level as the reference), cohabitation (with cohabiting as the reference), and wealth (with high level as the reference).

Furthermore, to evaluate possible effect modification (thus, to evaluate whether the observed overall association varies across patient subgroups) on a multiplicative and additive scale, the adjusted PRs and crude prevalence differences for continued opioid use were calculated while stratifying on sex, age, and comorbidities (CCI and preoperative psychiatric medication and NSAID use). PRs were adjusted for age when stratified by sex, for sex when stratified by age, and for sex and age when stratified by comorbidity. We evaluated both the direction and magnitude of the associations in strata compared with the associations from the overall analyses, focusing on clinically relevant interaction. In addition, we tested for statistical interaction by including interaction terms between potential effect modifiers and SES markers in the log-binominal regression model and calculating RR and P values for interaction terms. P values of < 0.05 were considered as statistically significant.

All results were based on the total number of patients at risk at the start of the examined time interval and calculated with 95% confidence intervals (CIs). Furthermore, all analyses were calculated for preoperative opioid non-users and users separately. The analyses were performed using Stata 17.0 (StataCorp LLP, College Station, TX, USA) and presented according to the Strengthening the Reporting of Observational Studies in Epidemiology (STROBE) guidelines for cohort studies.

### Missing data

**Figure 1:**
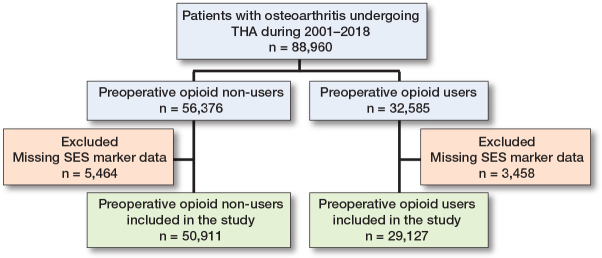
Flowchart. THA = primary total hip arthroplasty, SES = socioeconomic status.

All included patients were OA patients who underwent THA. Patients without complete data on SES markers were excluded (Figure 1). As patients with no redeemed prescriptions for opioids were considered non-users, there were no missing outcome data to report in this study. Data on patient characteristics were complete.

### Ethics, registration, funding, and disclosures

According to Danish law, ethics committee approval is not required for registry-based studies. The study was reported to the Danish Data Protection Agency through registration at Aarhus University (record number: AU-2016-051-000001, sequential number 880). All data generated or analyzed during this study is included in this published article (and its supplementary information files). According to the Danish legislation, datasets generated and/or analyzed during the current study are not publicly available. The first author received a grant from the Department of Clinical Medicine, Aarhus University Research foundation for covering salary expences.. The Department of Clinical Epidemiology at Aarhus University is involved in studies with funding from various pharmaceutical companies as institutional research grants to Aarhus University. These companies had no role in the study design, analysis and interpretation of data, writing of the manuscript, or decision to submit the manuscript for publication. The authors declare no conflicts of interest. Complete disclosure of interest forms according to ICMJE are available on the article page, doi: 10.2340/17453674.2024.40708

## Results

We found 88,960 patients undergoing primary THA for OA during 2001–2018 but 8,922 had missing SES marker data and were excluded. We thus included 80,038 patients in this study, of whom 50,911 were preoperative opioid non-users and 29,127 were preoperative users (Figure 1). Among preoperative non-users, the median age was 69 years, 52% were female, and 74% had a low CCI score. We found that 17% used preoperative psychiatric medication and 51% used NSAIDs. Among preoperative users, median age was 71 years, 62% were female, 61% had a low CCI score, 34% used preoperative psychiatric medication, and 62% used NSAIDs. Approximately 1–2% of patients died within 1 year after THA. Detailed patient characteristics stratified for SES markers are presented in Table 2.

**Table 2:** Patient characteristics of preoperative opioid non-users and users in relation to education, cohabitation, and wealth. Values are count (%)

Factor	Education			Cohabitation		Wealth		
	Low	Medium	High	Alone	Cohabiting	Low	Medium	High
**Preoperative non-users**	20,151	21,045	9,715	15,799	35,112	16,971	16,970	16,970
Female sex	11,609 (58)	9,615 (46)	5,462 (56)	11,083 (70)	15,603 (44)	10,348 (61)	8,448 (50)	7,890 (46)
Age								
46	190 (1)	613 (3)	252 (3)	259 (2)	796 (2)	279 (2)	386 (2)	390 (2)
46–55	1,027 (5)	2,464 (12)	1,023 (11)	980 (6)	3,534 (10)	1,114 (6)	1,410 (8)	1,990 (12)
56–65	3,873 (19)	6,288 (30)	2,923 (30)	2,784 (17)	10,300 (29)	4,694 (28)	4,499 (27)	3,891 (23)
66–75	8,766 (44)	8,172 (39)	3,819 (39)	6,107 (39)	14,650 (42)	6,353 (37)	7,023 (41)	7,381 (43)
> 75	6,295 (31)	3,508 (16)	1,698 (17)	5,669 (36)	5,832 (17)	4,531 (27)	3,652 (22)	3,318 (20)
Comorbidities (CCI)								
0	14,380 (71)	15,665 (75)	7,457 (77)	11,213 (71)	26,289 (75)	11,869 (70)	12,700 (75)	12,933 (76)
1–2	4,802 (24)	4,519 (21)	1,932 (20)	3,828 (24)	7,425 (21)	4,222 (25)	3,611 (21)	3,420 (20)
≥ 3	969 (5)	861 (4)	326 (3)	758 (5)	1,398 (4)	880 (5)	659 (4)	617 (4)
Medication								
Psychiatric	3,857 (19)	3,126 (15)	1,523 (16)	3,614 (23)	4,892 (14)	3,682 (22)	2,593 (15)	2,231 (13)
NSAIDs	10,460 (52)	10,707 (51)	4,681 (48)	8,029 (51)	17,819 (51)	9,025 (53)	8,794 (52)	8,029 (47)
**Preoperative users**	13,473	11,339	4,315	11,271	17,856	9,709	9,709	9,709
Female sex	9,056 (67)	6,172 (54)	2,900 (67)	8,749 (78)	9,379 (53)	6,931 (71)	5,864 (60)	5,333 (55)
Age								
< 46	101 (1)	208 (2)	57 (1)	119 (1)	247 (1)	123 (1)	102 (1)	141 (1)
46–55	609 (5)	1,051 (9)	359 (8)	574 (5)	1,445 (8)	522 (6)	619 (6)	878 (9)
56–65	2,476 (18)	3,162 (28)	1,193 (28)	1,934 (17)	4,897 (27)	2,360 (24)	2,371 (25)	2,100 (22)
66–75	5,523 (41)	4,380 (39)	1,726 (40)	4,052 (36)	7,577 (43)	3,598 (37)	3,905 (40)	4,126 (43)
> 75	4,764 (35)	2,538 (22)	980 (23)	4,592 (41)	3,690 (21)	3,106 (32)	2,712 (28)	2,464 (25)
Comorbidities (CCI)								
0	7,858 (58)	7,165 (63)	2,840 (66)	6,513 (58)	11,350 (64)	5,504 (57)	6,030 (62)	6,329 (65)
1–2	4,307 (32)	3,219 (28)	1,156 (27)	3,607 (32)	5,075 (28)	3,151 (32)	2,887 (30)	2,644 (27)
≥ 3	1,308 (10)	955 (9)	319 (7)	1,151 (10)	1,431 (8)	1,054 (11)	792 (8)	736 (8)
Medication								
Psychiatric	4,910 (36)	3,515 (31)	1,471 (34)	4,608 (41)	5,288 (30)	3,976 (41)	3,130 (32)	2,790 (29)
NSAIDs	8,047 (60)	7,087 (63)	2,802 (65)	6,636 (59)	11,300 (63)	5,745 (59)	6,061 (62)	6,130 (63)

Median wealth presented in dollars (non-users): income (low: 49,200; medium: 88,700; high: 135,200), liquid assets (low: 35,900; medium: 222,000; high: 529,800). Median wealth (users): income (low: 40,600; medium: 74,400; high: 118,400), liquid assets (low: 7,000; medium: 164,900; high: 432,400). Valuta exchange rate: 2018 average. CCI: Charlson Comorbidity Index.

### Prevalence of opioid use 

**Figure 2:**
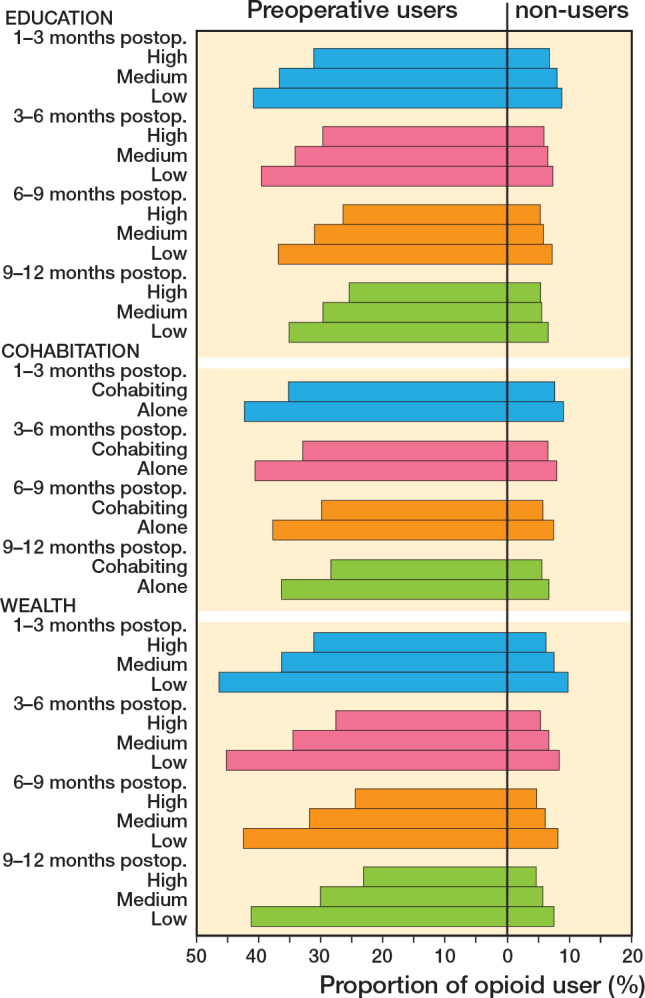
Prevalence of opioid use 1–12 months after THA among preoperative opioid users (left side) and non-users (right side) before THA, in relation to education, cohabitation, and wealth. 1–3 months after THA is depicted in blue, 3–6 in magenta, 6–9 in orange, and 9–12 in green.

Prevalences within 1 month after THA varied from 42% to 47% among preoperative non-users and from 60% to 64% among preoperative users for different SES markers (Tables 3 and 4). Prevalences for 1–3, 3–6, 6–9, and 9–12 months after THA are presented in Figure 2 (see Appendix). As prevalences for 1–3, 3–6, and 6–9 months were rather similar to prevalences for 9–12 months, we describe here in more details only those for 9–12 months.

**Table 3:** Opioid use 1 year after primary total hip arthroplasty among preoperative opioid non-users

Exposure	Patients at risk	Opioid users n (%)	Prevalence difference in percentage points		Prevalence ratio	
			crude (CI)	adjusted (CI) ^a^	crude (CI)	adjusted (CI) ^a^
Education						
Within 1 month						
High	9,715	4,494 (46)	0	0	1	1
Medium	21,045	9,980 (47)	1.2 (0.0 to 2.4)	1.2 (0.0 to 2.4)	1.03 (1.00 to 1.05)	1.03 (1.00 to 1.05)
Low	20,151	8,502 (42)	–4.1 (–5.3 to –2.9)	–3.0 (–4.2 to –1.8)	0.91 (0.89 to 0.94)	0.93 (0.91 to 0.96)
1–12 months						
High	4,494	(21)	0	0	1	1
Medium	9,980	(23)	2.0 (0.5 to 3.4)	2.8 (1.3 to 4.2)	1.09 (1.02 to 1.17)	1.13 (1.05 to 1.20)
Low	8,502	(28)	6.3 (4.8 to 7.8)	6.1 (4.6 to 7.6)	1.30 (1.21 to 1.38)	1.28 (1.20 to 1.37)
Cohabiting status						
Within 1 month						
Cohabiting	35,112	16,021 (46)	0	0	1	1
Alone	15,799	6,955 (44)	–1.6 (–2.5 to –0.7)	–0.8 (–1.8 to 0.2)	0.96 (0.94 to 0.99)	0.99 (0.96 to 1.01)
1–12 months						
Cohabiting	16,021	(23)	0	0	1	1
Alone	6,955	(27)	3.9 (2.7 to 5.1)	2.3 (1.0 to 3.6)	1.17 (1.11 to 1.23)	1.09 (1.04 to 1.15)
Wealth						
Within 1 month						
High	16,970	8,045 (47)	0	0	1	1
Medium	16,970	7,529 (44)	–3.0 (–4.1 to –2.0)	–2.9 (–4.0 to –1.9)	0.94 (0.91 to 0.96)	0.94 (0.92 to 0.96)
Low	16,971	7,402 (44)	–3.8 (–4.9 to –2.7)	–3.6 (–4.6 to –2.5)	0.92 (0.90 to 0.94)	0.92 (0.90 to 0.95)
1–12 months						
High	8,045	(20)	0	0	1	1
Medium	7,529	(24)	4.2 (2.9 to 5.5)	3.9 (2.6 to 5.2)	1.21 (1.14 to 1.28)	1.20 (1.13 to 1.27)
Low	7,402	(30)	9.6 (8.2 to 10.9)	8.7 (7.3 to 10.1)	1.48 (1.40 to 1.56)	1.43 (1.35 to 1.51)

Patients at risk during 1–12 months are those who were opioid users within 1 month and alive at the beginning of the 1–12 months period. However, to follow the Danish legislation concerning person-identifiable data and cope with a small number of patients who died within 1 month, we masked these numbers (in some categories) and further only reported rounded prevalences for this interval.

a adjusted for sex and age. CI = 95% confidence interval.

**Table 4:** Opioid use 1 year after primary total hip arthroplasty among preoperative opioid users

Exposure	Patients at risk	Opioid users n (%)	Prevalence difference in percentage points		Prevalence ratio	
			crude (CI)	adjusted (CI) ^a^	crude (CI)	adjusted (CI) ^a^
Education						
Within 1 month						
High	4,315	2,702 (63)	0	0	1	1
Medium	11,339	7,159 (63)	0.5 (–1.2 to 2.2)	0.7 (–1.0 to 2.4)	1.01 (0.98 to 1.04)	1.01 (0.98 to 1.04)
Low	13,473	8,291 (62)	–1.1 (–2.7 to 0.6)	–0.2 (–1.9 to 1.4)	0.98 (0.96 to 1.01)	1.00 (0.97–1.02)
1–12 months						
High	2,702	(55)	0	0	1	1
Medium	7,159	(61)	5.8 (3.6 to 8.0)	7.1 (4.9 to 9.3)	1.10 (1.06 to 1.15)	1.13 (1.09 to 1.18)
Low	8,291	(67)	12.2 (10.0 to 14.3)	12.0 (9.9 to 14.1)	1.22 (1.18 to 1.27)	1.22 (1.17 to 1.27)
Cohabiting status						
Within 1 month						
Cohabiting	17,856	11,116 (62)	0	0	1	1
Alone	11,271	7,036 (62)	0.2 (–1.0 to 1.3)	0.9 (–0.3 to 2.1)	1.00 (0.98 to 1.02)	1.02 (1.00 to 1.04)
1–12 months						
Cohabiting	11,116	(60)	0	0	1	1
Alone	7,036	(68)	8.3 (6.9 to 9.7)	6.1 (4.6 to 7.6)	1.14 (1.11 to 1.16)	1.10 (1.07 to 1.12)
Wealth						
Within 1 month						
High	9,709	6,064 (62)	0	0	1	1
Medium	9,709	5,834 (60)	–2.4 (–3.7 to –1.0)	–2.2 (–3.6 to (0.8)	0.96 (0.94 to 0.98)	0.96 (0.94 to 0.99)
Low	9,709	6,254 (64)	2.0 (0.6 to 3.3)	2.1 (0.8 to 3.5)	1.03 (1.01 to 1.05)	1.03 (1.01 to 1.06)
1–12 months						
High	6,064	(54)	0	0	1	1
Medium	5,834	(62)	8.1 (6.4 to 9.9)	7.6 (5.9 to 9.4)	1.15 (1.12 to 1.19)	1.14 (1.11 to 1.18)
Low	6,254	(73)	18.9 (17.2 to 20.6)	17.4 (15.7 to 19.1)	1.35 (1.31 to 1.39)	1.32 (1.28 to 1.36)

See footnote Table 2.

For preoperative non-users, the prevalence of opioid use in 9–12 months ranged from 4.8% for patients with high education to 6.2% for patients with low education, whereas among preoperative users, these prevalences were 24.4% and 33.8%, respectively (Figure 2, see Appendix). Among preoperative non-users, the prevalence was 5.1% for patients who were cohabiting and 6.3% for patients living alone, compared with 27.4% and 35.0%, respectively, among preoperative users (Figure 2, see Appendix). For preoperative non-users, prevalences ranged from 4.4% for patients with high wealth to 6.9% for low wealth, compared with 22.3% and 39.6%, respectively, among preoperative users (Figure 2, see Appendix).

### Prevalence ratios and prevalence differences

The PRs for immediate opioid use within 1 month after THA showed little to no difference within the 3 SES markers, in both preoperative non-users and users (Tables 3 and 4). Among 22,976 preoperative non-users who were immediate opioid users, adjusted PRs for continued opioid use were 1.13 (CI 1.05–1.20) for medium education and 1.28 (CI 1.20–1.37) for low education compared with patients who had high education; 1.09 (CI 1.04–1.15) for patients living alone compared with patients who were cohabiting; and 1.20 (CI 1.13–1.27) for medium wealth and 1.43 (CI 1.35–1.51) for low wealth compared with patients who had high wealth (Table 3). Among the 18,152 preoperative users who were immediate users, adjusted PRs for continued opioid use were 1.13 (CI 1.09–1.18) for medium education and 1.22 (CI 1.17–1.27) for low education compared with patients having high education; 1.10 (CI 1.07–1.12) for patients living alone compared with those who were cohabiting; and 1.14 (CI 1.11–1.18) for medium wealth and 1.32 (CI 1.28–1.36) for low wealth compared with patients who had high wealth (Table 4).

Crude prevalence differences of continued opioid use were considerably lower overall among preoperative non-users than among users. The largest prevalence difference (in percentage points) among non-users was in patients with low wealth at 9.6 (CI 8.2–10.9) compared with patients with high wealth. The corresponding prevalence difference was 18.9 (CI 17.2–20.6) in preoperative opioid users (Tables 3 and 4).

### Sex, age, and comorbidities as effect measure modifiers

**Figure 3:**
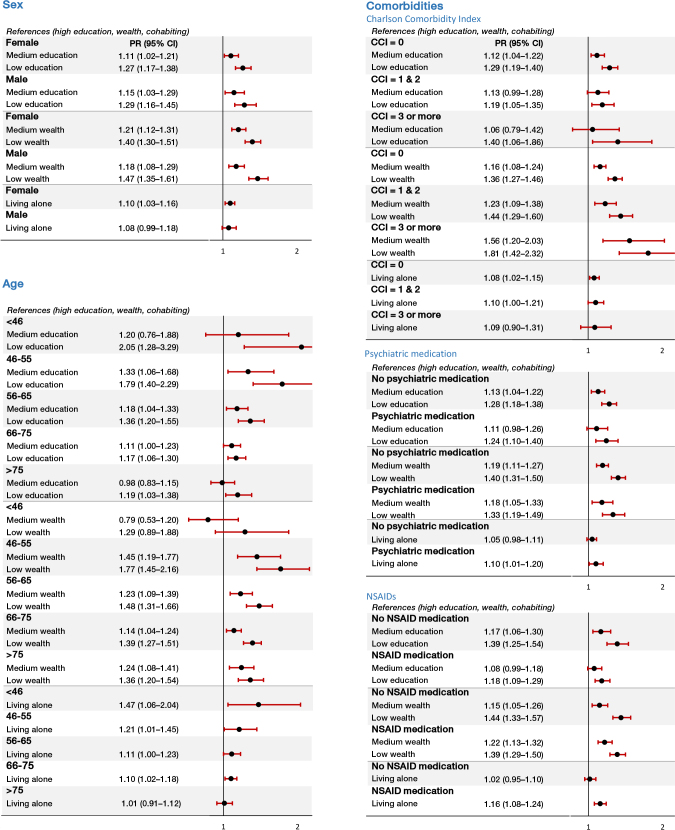
Opioid use after primary THA among preoperative opioid non-users, in relation to SES markers stratified by sex, age, and comorbidities. THA: total hip arthroplasty; PR: adjusted prevalence ratio: sex (adjusted for age), age (adjusted for sex), comorbidities (adjusted for sex and age); CI: confidence interval; CCI: Charlson Comorbidity Index. Opioid use is defined as having redeemed at least 1 prescription for opioids 1–12 months after THA

**Figure 4:**
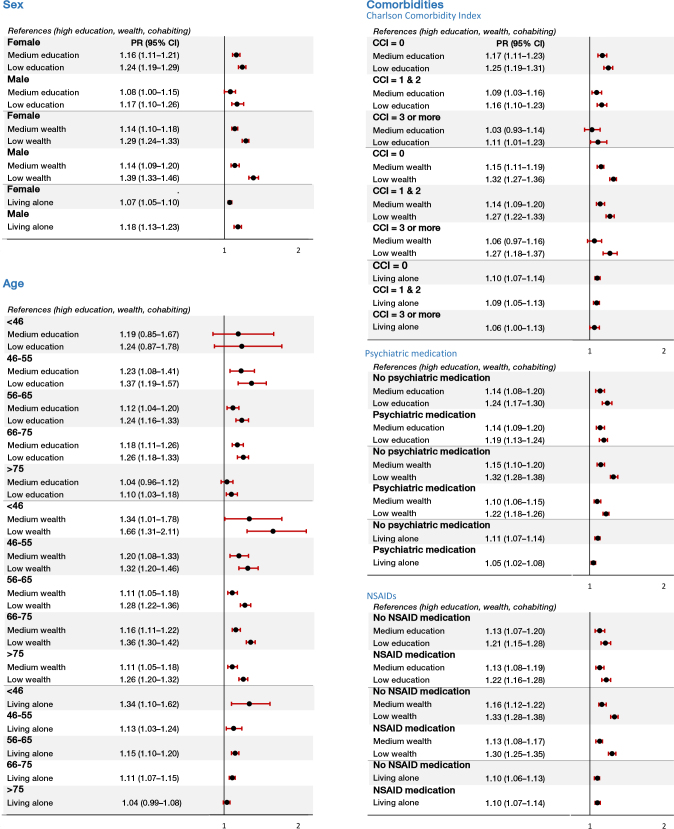
Opioid use after primary THA among preoperative opioid users in relation to SES markers stratified by sex, age, and comorbidities. THA: total hip arthroplasty; PR: adjusted prevalence ratio: sex (adjusted for age), age (adjusted for sex), comorbidities (adjusted for sex and age); CCI: Charlson Comorbidity Index. Opioid use is defined as redeeming at least 1 prescription for opioids 1–12 months after THA.

In general, crude prevalence differences and adjusted PRs of continued opioid use in the stratified analyses had similar direction of the association, as in the overall analyses (Figures 3 and 4). The association between all SES markers and continued opioid use was stronger in younger patients than in older patients. In preoperative non-users, the adjusted PRs for low vs. high education were 2.05 (CI 1.28–3.29) in younger (< 46) and 1.19 (CI 1.03–1.38) in older (> 75) patients. In addition, the crude prevalence difference was 16.0 (CI 5.5–26.5) comparing low vs. high education in younger patients, whereas the prevalence difference was 4.7 (CI 1.1–8.3) among older patients. Nevertheless, test for interaction showed that the association between SES markers and opioid use was not statistically significant different across the levels of sex, age, and comorbidity (all P values for interaction terms were > 0.05).

## Discussion

We aimed to examine the association between SES markers and opioid use after primary THA for OA and whether sex, age, or comorbidities modify any association. Based on 80,038 patients undergoing primary THA for OA, we found that low education, living alone, and low wealth were associated with a higher prevalence of continued use of opioids 1–12 months after THA. Markers of low SES did not have any effect on immediate opioid use within 1 month after THA. We also found that association between SES markers and continued opioid use was stronger in younger than in older patients. Results are clinically relevant as they may help health professionals at hospital and in primary care focus on socially vulnerable THA patients and explore possible future interventions aiming at reducing the social gradient in THA opioid use and thereby improving THA outcome.

It has been reported that preoperative users are at much higher risk of postoperative use than preoperative non-users [15,16]. Therefore, we conducted our analyses on preoperative opioid non-users and users separately. In a US study, Goesling et al. reported that 4% of preoperative non-users and 35% of preoperative users were using opioids 6 months after hip and knee arthroplasty [15], whereas Rajamäki et al. reported that 41% of preoperative analgesic users were using opioids 9–12 months after hip and knee arthroplasty in Finland [16]. These prevalences align with those we observed in the current work. Preoperative opioid users are likely to be treated for diagnoses other than OA because opioid treatment is not recommended for treating OA-related pain. Reporting prevalences separately for preoperative non-users and users is highly clinically relevant because postoperative interventions with the aim of reducing opioid use differ between preoperative users and non-users due to different indications for preoperative treatment. However, we cannot entirely exclude the possibility that clinical guidelines are not always followed and that some patients have received preoperative opioid prescriptions for OA-related pain, particularly if they are close to THA surgery.

Our result showing no association between SES and immediate opioid within 1 month after THA aligns with our recent findings of a substantial increase since 1995 in opioid use in the acute postsurgical phase, and now applies to the majority of patients after THA as a common element of post-surgical treatment [17]. More aggressive early and timely pain treatment with opioids in all patients is highly relevant to promoting rehabilitation and does not seem to affect continued opioid use [17]. However, our study was conducted on Danish patients, and it is important to note that other studies have found higher doses of acute postoperative opioid use to be associated with prolonged opioid use [18,19]. To our knowledge, no other study has examined the relationship between SES and immediate opioid use among THA patients.

Our findings regarding the impact of SES on continuous opioid use are in line with studies showing an association between markers of low SES, such as education and wealth, and postsurgical pain. Feldman et al. [20] found that low education and area-based SES are associated with higher levels of pain after total knee arthroplasty. Mesterton et al. found similar associations in a THA cohort in Sweden, showing that patients with high education and income reported lower pain levels after surgery than patients who had low education and income [21].

We did not find that age modified the effect of SES to the extent of an inverse or opposite association, neither did we find statistically significant interaction between age and SES. However, the association between low SES and continued opioid use was stronger in young compared with old patients. Some evidence indicates that younger age at THA is a risk factor for persistent opioid use [22] and that younger THA patients use higher doses of opioids perioperatively [23]. Age has previously been reported as an effect modifier of the association between education and income and the risk of revision surgery after THA [24]. Return to work is an important rehabilitation goal for younger patients, most of whom are active on the labor market, so younger patients may be more willing to take opioids to speed up rehabilitation and return to work. In addition, over the last few decades, there has been a considerable change in educational opportunities so that older people are overrepresented among patients with less education, which could have affected our results. Furthermore, social support is an important factor in dealing with physical and psychological stress [25]. Older patients living alone might be less dependent on social support because healthcare professionals are often responsible for administering their medicine at home. This fits with our finding showing associations that were slightly stronger for living alone vs. cohabiting in younger than in older patients.

### Limitations

We did not have data on the specific indication for the opioid prescription. We tried to account for this gap by analyzing data separately for preoperative non-users and users. Opioid prescriptions after THA among preoperative non-users are more likely to be related to the THA procedure than to treatment for an underlying chronic condition, as might be the case for preoperative users. We lack information on other surgical procedures, but to avoid interference with the rehabilitation program, surgeries are seldom planned within 1 year of primary THA. Although we included sex, age, comorbidity, and psychiatric medication and NSAID use, several other factors could potentially explain the observed association. For example, patients with basic education or low income have higher odds of inadequate health literacy [26], which can play a crucial role in motivation and competence to access and understand health information. Patients with low SES more often have a poor social network or low social support [27], which could affect their ability to engage in rehabilitation. Furthermore, lifestyle measures add other important aspects to further improve knowledge regarding social inequity in health.

### Conclusions

Low education level, living alone, and low wealth were associated with a higher prevalence of continued use of opioids 1–12 months after THA. Age modified the magnitude but not the direction of the effect of SES on the risk of opioid use.

## Notes

All authors contributed substantially to the intellectual content including the design of the study, interpretation of data, critical revision of the manuscript, and final approval of the submitted version. ASK contributed to the statistical analyses and drafted and revised the manuscript.

Handling co-editors: Keijo Mäkelä and Philippe Wagner

Acta thanks Enrico De Visser for help with peer review of this manuscript.
